# Biotinylation of the *Neospora caninum* parasitophorous vacuole reveals novel dense granule proteins

**DOI:** 10.1186/s13071-021-05023-7

**Published:** 2021-10-09

**Authors:** Congshan Yang, Chenrong Wang, Jing Liu, Qun Liu

**Affiliations:** 1 National Animal Protozoa Laboratory, College of Veterinary Medicine, China Agricultural University, Beijing, China; 2 College of Animal Science and Technology, Anhui Agricultural University, Hefei, Anhui 230036 China

**Keywords:** *Neospora caninum*, Dense granule protein, BioID, Parasitophorous vacuole, CRISPR/Cas9

## Abstract

**Background:**

*Neospora caninum* is an obligate intracellular parasite that invades host cells and replicates within the parasitophorous vacuole (PV), which resists fusion with host cell lysosomal compartments. To modify the PV, the parasite secretes an array of proteins, including dense granule proteins (GRAs). The vital role of GRAs in the *Neospora* life cycle cannot be overestimated. Despite this important role, only a subset of these proteins have been identified, and most of their functions have not been elucidated. Our previous study demonstrated that NcGRA17 is specifically targeted to the delimiting membrane of the parasitophorous vacuole membrane (PVM). In this study, we utilize proximity-dependent biotin identification (BioID) to identify novel components of the dense granules.

**Methods:**

NcGRA17 was BirA* epitope-tagged in the Nc1 strain utilizing the CRISPR/Cas9 system to create a fusion of NcGRA17 with the biotin ligase BirA*. The biotinylated proteins were affinity-purified for mass spectrometric analysis, and the candidate GRA proteins from BioID data set were identified by gene tagging. To verify the biological role of novel identified GRA proteins, we constructed the *NcGRA23* and *NcGRA11 (a–e)* knockout strains using the CRISPR/Cas9 system and analyzed the phenotypes of these mutants.

**Results:**

Using NcGRA17-BirA* fusion protein as bait, we have identified some known GRAs and verified localization of 11 novel GRA proteins by gene endogenous tagging or overexpression in the Nc1 strain. We proceeded to functionally characterize NcGRA23 and NcGRA11 (a–e) by gene knockout. The lack of NcGRA23 or NcGRA11 (a–e) did not affect the parasite propagation in vitro and virulence in vivo.

**Conclusions:**

In summary, our findings reveal that BioID is effective in discovering novel constituents of *N. caninum* dense granules. The exact biological functions of the novel GRA proteins are yet unknown, but this could be explored in future studies.

**Graphical abstract:**

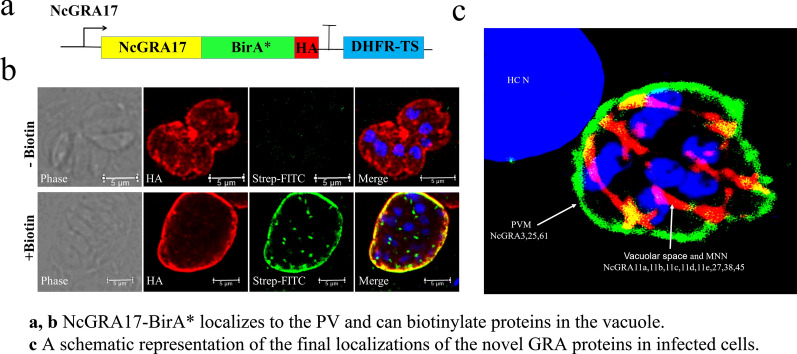

**Supplementary Information:**

The online version contains supplementary material available at 10.1186/s13071-021-05023-7.

## Background

*Neospora caninum*, a member of the phylum Apicomplexa, is an obligate intracellular parasite that causes neosporosis, with worldwide distribution [1]. The parasite has been recognized as one of the most important infectious causes of reproductive issues and abortion in cattle, and 12–42% of aborted bovine fetuses worldwide are infected [2, 3]. Given the high prevalence of the parasite, neosporosis is a major threat facing dairy and livestock industries and results in significant economic losses [3, 4].

*Neospora caninum* has three specific secretory organelles, namely micronemes, rhoptries, and dense granules, which sequentially discharge effectors during invasion. The dense granule (GRA) proteins are essential in the formation of parasitophorous vacuoles (PVs) within which the parasites multiply and grow [5, 6]. There are three localizations in PVs within the infected cell where *Neospora* dense granule proteins (NcGRAs) are exhibited: the vacuolar space, intravacuolar membranous nanotubular network (MNN), and parasitophorous vacuole membrane (PVM). Although the dense granule proteome is hypothesized to be composed of hundreds of proteins, only nine GRA proteins (NcGRA1, NcGRA2, NcGRA6, NcGRA7, NcGRA9, NcGRA12, NcGRA14, NcGRA17, and NcGRA23) [7–15] have been previously identified in *N. caninum*; however, the precise roles of these GRA proteins in the intracellular survival and growth of the parasites are not well elucidated.

To date, most NcGRAs have been discovered individually by bioinformatics searches for proteins exhibiting homology to known *Toxoplasma* dense granule proteins (TgGRAs). Although the bioinformatics approach has been successful, we sought a method to identify a large number of dense granule components more rapidly. In this study, we adapted the proximity-dependent biotin identification (BioID) system for *Neospora* and used the dense granule protein NcGRA17 as bait to identify novel NcGRA proteins. We demonstrate that the NcGRA17-BirA* fusion localizes accurately to the dense granules, is catalytically active, and labels previously described NcGRA proteins as well as novel targets. A number of these targets were confirmed by gene overexpression or endogenous tagging in the Nc1 strain, thereby validating our approach. Subsequently, six of them were characterized by gene disruption. The discovery of novel NcGRA proteins provides a more comprehensive basis for the understanding of host–parasite interactions and *N. caninum* parasitism.

## Methods

### Parasites and cell cultures

All *Neospora* tachyzoites used in this study were propagated in a human foreskin fibroblast (HFF) line cultured in complete Dulbecco’s modified Eagle’s medium (DMEM) supplemented with 10% fetal bovine serum (FBS, Gibco, USA), as described previously [14, 16]. Both cells and parasites were incubated at 37°C with 5% CO_2_ in a humidified incubator.

### Plasmid construction

All primer sequences are shown in Additional file 3: Table S1. The *NcGRA17*-targeting CRISPR plasmid (pNc_Cas9CRISPR::sgNcGRA17) was constructed as described previously [14]. All other CRISPR/Cas9 plasmids were obtained through FastPfu (TansGen Biotech Co., Ltd., China) mutagenesis of the above plasmid to change the *NcGRA17* targeting gRNA to other specific gRNAs. To generate clean knockouts of *NcGRA11 (a-e)* genes (62240 bp), a double-gRNA CRISPR/Cas9 system was designed in which the first gRNA sequence (gRNA1) was placed close to the start codon of the first gene (*NcGRA11a*), with the second gRNA (gRNA2) near the stop codon of the last gene (*NcGRA11e*), as described previously [17]. To express two gRNAs from a single plasmid pNc_Cas9CRISPR::sgNcGRA11(a–e), the gRNA2 expression cassette (NcU6 promoter-gRNA2-RNA scaffold TTTT) was amplified using a common set of primers, 2×gRNA NcU6 F and 2×gRNA NcU6 R, and cloned into the pNc_Cas9CRISPR::sgNcGRA11(a–e)^1^ plasmid using a one-step cloning kit (Vazyme Biotech Co., Ltd., China). For disruption of *NcGRA11 (a–e)* genes, CRISPR/Cas9 double-gRNA plasmid pNc_Cas9CRISPR::sgNcGRA11(a–e) was co-transfected with its corresponding DHFR-TS amplicon containing 60-bp homology regions matching the *NcGRA11 (a–e)* genes. To generate a construct (pLIC-BirA*-HA-DHFR-NcGRA17) for inserting a BirA*-HA tag into the *NcGRA17* gene 3′ end, upstream (830-bp) and downstream (950-bp) regions directly adjacent to the gRNA target sequence and BirA*(963 bp) sequence were cloned into the pLIC-HA vector. pDMG plasmid [16, 18, 19] was used to express *NcGRA11 (a-e)* genes in the Nc1 strain. The coding sequences of *NcGRA11 (a–e)* were cloned into the backbone plasmid pDMG with GFP replaced by HA [18] using a one-step cloning kit (Vazyme Biotech Co., Ltd., China). The vector p6 × HA-HXGPRT used for in situ C-terminal tagging, as described previously [20], was generously provided by Prof. Shaojun Long of China Agricultural University. This plasmid was modified by replacing the 6HA tag with the spaghetti monster tag smHA [21] and replacing the selectable marker HXGPRT with DHFR. The construct (pTCR-NcGRA23-CD KO) for disrupting the *NcGRA23* locus was designed by inserting the 5′ (990-bp) and 3′ (930-bp) regions flanking the *NcGRA23* gene region into the backbone plasmid pTCR-CD, which has been described previously [18].

### Generation of NcGRA17-BirA* fusions

C-terminal BirA*-HA-tagging of NcGRA17 was performed via homologous recombination. Nc1, as the parent strain, was co-transfected with the pNc_Cas9CRISPR::sgNcGRA17 vector and homologous repair amplicon. The amplicon was amplified using primers listed in Additional file 3: Table S1 and pLIC-BirA*-HA-DHFR-NcGRA17 plasmid as a template. The parasites were cultured to the third generation in the presence of pyrimethamine (1 µM) and then screened to confirm the purity of the selected strains. The selected NcGRA17-tagged strain, named NcGRA17-BirA*-HA, was identified by polymerase chain reaction (PCR), western blotting, and immunofluorescence assay.

### Affinity capture of biotinylated proteins

BirA*-tagged and parent lines were used to infect HFF monolayers and grown in medium containing 150 µM biotin for 48 h prior to parasite egress [22]. Infected cells were collected, washed in phosphate-buffered saline (PBS), and lysed in radioimmunoprecipitation assay (RIPA) buffer (Beyotime, China) supplemented with protease inhibitor cocktail (Sigma-Aldrich, USA) for 30 min on ice. Lysates were centrifuged for 15 min at 14,000×*g* to pellet insoluble debris and then incubated with streptavidin magnetic beads (Beaver, China) at room temperature for 4 h under gentle agitation. Beads were collected using magnets and washed five times in RIPA buffer, followed by three washes in 8 M urea buffer (50 mM Tris–HCl [pH 7.4], 150 mM NaCl). Ten percent of each sample was boiled in Laemmli sample buffer, and eluted proteins were analyzed by western blotting with streptavidin-horseradish peroxidase (HRP) before mass spectrometry (MS) analysis.

### Mass spectrometry

Purified proteins bound to streptavidin beads were reduced, alkylated, and digested by the sequential addition of Lys-C and trypsin proteases [22]. The peptide mixture was desalted using C18 tips and fractionated by a 75 µm inner diameter filter fused to the silica capillary column with a 5 µm pulled electrospray tip, and packed in-house with 15 cm of a Luna C18 column with 3 µm reversed-phase particles. Delivery of the gradient was performed by the EASY-nLC 1000 ultrahigh-pressure liquid chromatography (UHPLC) system (Thermo Scientific). Tandem mass spectrometry (MS/MS) spectra were collected on a Q Exactive mass spectrometer (Thermo Scientific). Data analysis was performed using ProLuCID and DTASelect2 implemented in the Integrated Proteomics Pipeline (IP2) platform (Integrated Proteomics Applications, Inc., San Diego, CA, USA). Protein and peptide identification was filtered using DTASelect and required a minimum of two unique peptides per protein and a peptide-level false-positive rate of less than 5%, as estimated by a decoy database strategy. Normalized spectral abundance factor (NSAF) values were calculated as described previously [22].

### Generation of NcGRA-HA parasites

As few data are available from *Neospora*, we utilized homologous gene expression data of *Toxoplasma* to filter hits from our BioID data set for further investigation. To epitope-tag the candidate NcGRA proteins, CRISPR/Cas9-sgNcGRA plasmid was co-transfected with their corresponding smHA tag amplicon containing 42-bp homology regions matching the gene of interest. The amplicon was amplified using primers listed in Additional file 3: Table S1 and psmHA-DHFR plasmid as a template. As we failed to endogenous HA-tag the *NcGRA11b* gene, the vector pDMG-NcGRA11b-HA was electroporated into Nc1. The transfected parasites were grown in medium containing 1 µM pyrimethamine, and identified by immunofluorescence assay (IFA). All confirmed NcGRAs were examined in silico using the Basic Local Alignment Search Tool (BLAST) to search for other *Neospora* proteins with sequence similarity.

### Deletion of the genes encoding NcGRA23 and NcGRA11 (a–e)

NcGRA23-KO parasites were generated via homologous recombination. The parent Nc1 strain was co-transfected with the pNc_Cas9CRISPR::sgNcGRA23 and linearized complete knockout plasmid pTCR-NcGRA23-CD KO (primers listed in Additional file 3: Table S1). The transgenic parasites were grown under chloramphenicol (20 µM) and 5-fluorine cytosine (40 µM) selection pressure. The selected *NcGRA23*-deficient strain, named ∆*ncgra23*, was identified by PCR and western blotting. For disruption of *NcGRA11 (a–e)* genes, CRISPR/Cas9 double-gRNA plasmid pNc_Cas9CRISPR::sgNcGRA11(a–e)^1–2^ was co-transfected with its corresponding DHFR-TS amplicon containing 60-bp homology regions matching the *NcGRA11 (a–e)* genes. The parasites were cultured in the presence of pyrimethamine (1 µM) to the third generation. Resistant clones were isolated and confirmed by PCR and western blotting.

### Western blotting

Western blotting was performed as described previously [14]. The parent and modified strains of whole-parasite lysates were resolved using sodium dodecyl sulfate-polyacrylamide gel electrophoresis (SDS-PAGE) on a 12% (w/v) gel. Samples were transferred onto polyvinylidene fluoride membranes and probed with the mouse anti-HA antibody (Sigma-Aldrich, 1:500), the mouse anti-NcGRA23 antibody (prepared in our laboratory, 1:200), or the mouse anti-NcGRA11c antibody (prepared in our laboratory, 1:200). The *N. caninum* F-actin subunit beta (NcActin) was used as a loading control and was incubated with the rabbit anti-NcActin antibody (prepared in our laboratory, 1:2000). For all secondary antibody incubations, HRP-labeled goat anti-mouse IgG (H+L) antibody (Sigma, USA) was diluted 1:5000, an HRP-labeled goat anti-rabbit IgG secondary antibody (Sigma, USA) was diluted 1:10,000, or streptavidin-HRP (4A Biotech Co., Ltd., China) was used at a 1:1000 dilution. Following secondary incubation, enhanced chemiluminescence reagents (CoWin, China) were used for the detection of HRP activity.

### Immunofluorescence assay

Immunofluorescence assay (IFA) to detect the biotinylated proteins of BirA*-tagged parasites was performed as described previously [22]. Parasites were used to infect HFF monolayers on coverslips in a 12-well plate and grown for 30 h with the addition of 150 μM biotin. Infected cells were fixed for 15 min in 4% formaldehyde, and then permeabilized with 0.25% Triton X-100 for 15 min and blocked with 3% bovine serum albumin (BSA) for 30 min. Subsequently, the cells were incubated with a rabbit anti-HA polyclonal antibody (CWBIOTECH, China, 1:50); then incubation was performed with Cy3-conjugated goat anti-rabbit IgG (H+L) (Sigma, USA) and streptavidin/fluorescein isothiocyanate (FITC) (Solarbio, 1:100). The images were obtained using a Leica confocal microscope system (Leica TCS SP52, Germany).

To analyze NcGRA localization, parasite-infected HFFs or freshly released parasites were processed for IFA and stained with a rabbit anti-HA polyclonal antibody, mouse anti-NcGRA6 polyclonal antibody (prepared in our laboratory, 1:50), or mouse anti-NcGRA23 polyclonal antibody (1:50). To determine the trafficking route of the protein following secretion into the PV, freshly harvested parasites (NcGRA17-HA or NcGRA11c-HA) were used to pulse-infect monolayers of HFF cells for 3 min at 37 °C. Monolayers were washed with PBS and further incubated at 37 °C for 5 min, 10 min, 15 min, or 1 h, and then fixed and permeabilized in 0.002% saponin [23]. Localization of the indicated NcGRAs was performed by double immunofluorescence labeling, using both the rabbit serum anti-HA and the mouse serum anti-NcGRA6 or anti-NcGRA23. Primary antibodies were revealed using FITC-conjugated goat anti-mouse IgG (H+L) and Cy3-conjugated goat anti-rabbit IgG (H+L). The IFA process and image collection were performed as described above.

### Plaque assay

Plaque assays were performed on HFF cells cultured in 12-well plates (Corning Costar, USA) as described previously [14, 16]. Briefly, 200 freshly isolated parasites were seeded into HFF monolayers and incubated at 37 °C with 5% CO_2_ for 9 days undisturbed. The plaques were scanned using a Canon digital scanner (model F917500, Japan), and the plaque area was measured as described previously [18].

### Parasite virulence assay

The virulence of the parasites was investigated as described previously [14]. Eight-week-old female BALB/c mice (Laboratory Animal Center of Academy of Military Medical Sciences, Beijing, China) were injected intraperitoneally with *∆ncgra23*, ∆*ncgra11(a–e)*, or parent Nc1 parasites at doses of 5 × 10^6^ and 8 × 10^6^ parasites (*n* = 5 mice/dose). Mice were carefully monitored every 8 h based on their clinical signs and mortality, and humanely euthanized via cervical dislocation when they were unable to reach food or water for more than 24 h or lost 20% of normal body weight. Survival was monitored for 60 days.

### Statistical analysis

All statistical data were analyzed using GraphPad Prism 5 (version 5.01; GraphPad Software, San Diego, CA, USA) except as otherwise indicated above. The results were expressed as mean ± SD and evaluated by non-parametric tests. Values of *P* < 0.05 and *P* < 0.01 were considered statistically significant.

## Results

### Biotinylation of the PV using NcGRA17 as bait

**Fig. 1:**
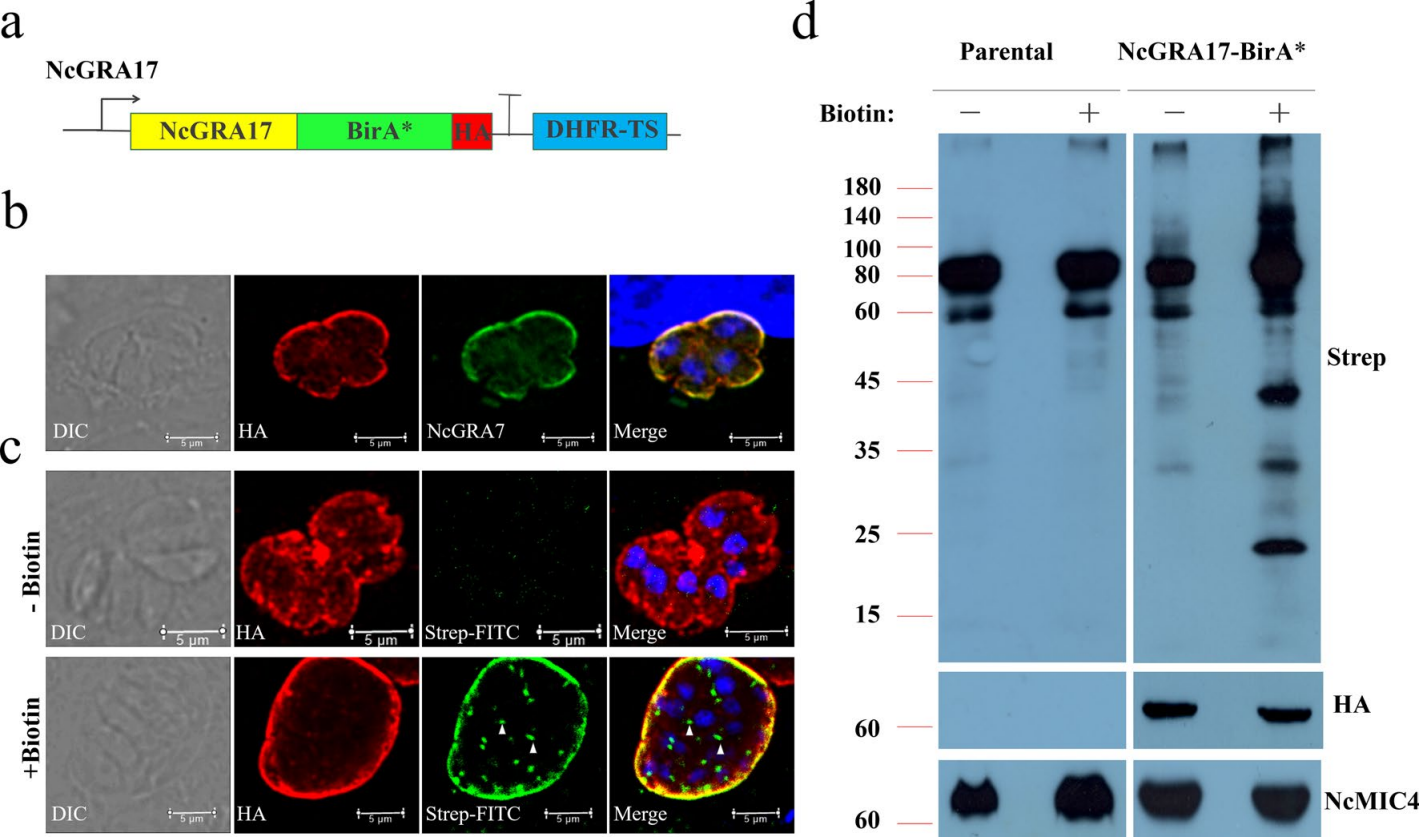
NcGRA17-BirA* localizes to the PV and can biotinylate proteins in the vacuole. **a** Diagram of the construct encoding the full genomic sequence of NcGRA17 fused to BirA* along with a 3 × HA C-terminal epitope tag. **b** IFA showing that NcGRA17-BirA* traffics appropriately to the PV and co-localizes with NcGRA7 (scale bar = 5 µm). **c** IFA of NcGRA17-BirA* expressing parasites ± biotin, showing the PV is labeled in a biotin-dependent manner. Endogenously biotinylated apicoplasts are observed with biotin (arrowheads) (scale bar = 5 µm). **d** Western blotting of whole-cell lysates of parent (Nc1) and NcGRA17-BirA*-expressing parasites ± biotin. Lysates were probed with streptavidin-HRP, revealing an increase in biotinylated proteins in NcGRA17-BirA*-expressing parasites upon the addition of biotin

To identify novel NcGRA proteins, we adapted the BioID approach using NcGRA17 as bait. NcGRA17 was BirA* epitope-tagged in the Nc1 strain utilizing the CRISPR/Cas9-directed genome editing system to create a fusion of NcGRA17 with the biotin ligase BirA* (Fig. 1a; Additional file 1: Figure S1a–c). The localization of the NcGRA17-BirA* fusion was assessed by IFA. As expected, we observed staining in the PV (Fig. 1b), a pattern consistent with NcGRA7, indicating that the fusion did not alter the trafficking of NcGRA17. To determine which proximal proteins in the PV were labeled by the NcGRA17-BirA* tachyzoites, we grew parasites in medium supplemented with biotin and stained for biotinylated proteins using fluorophore-conjugated streptavidin. NcGRA17-BirA* tachyzoites showed a robust streptavidin staining in the PV, indicating that the biotin ligase fusion was active and that it labeled proteins in the PV (Fig. 1c, bottom). We then assessed the labeling of *N. caninum* proteins by NcGRA17-BirA* in whole-cell lysates by western blotting with a streptavidin-HRP probe. There was a significant increase in biotinylated proteins in lysates of NcGRA17-BirA* parasites, and only when biotin was present in the growth medium (Fig. 1d). Collectively, these data demonstrate that NcGRA17-BirA* is active and biotinylates proteins in the PV.

### Identification of novel dense granule proteins

The target labeled was identified by NcGRA17-BirA* as we affinity-purified biotinylated proteins from parasite lysates for MuDPIT mass spectrometric analysis. Biotinylated proteins from both parent and NcGRA17-BirA* lysates were significantly enriched using streptavidin-conjugated magnetic beads. Proteins identified by MS that were unique to NcGRA17-BirA* samples were scored as hits based on the number of identified spectra and unique peptides (see Additional file 4: Table S2 and Additional file 5: Table S3). Compared to a control lysate derived from Nc1 supplemented with biotin, the NcGRA17-BirA* purified fraction revealed two known dense granule proteins: NcGRA2 and NcGRA23 [7, 8]. As few data are available from *Neospora*, we utilized homologous gene expression data of *Toxoplasma* to filter hits from our BioID data set for further investigation.

**Fig. 2:**
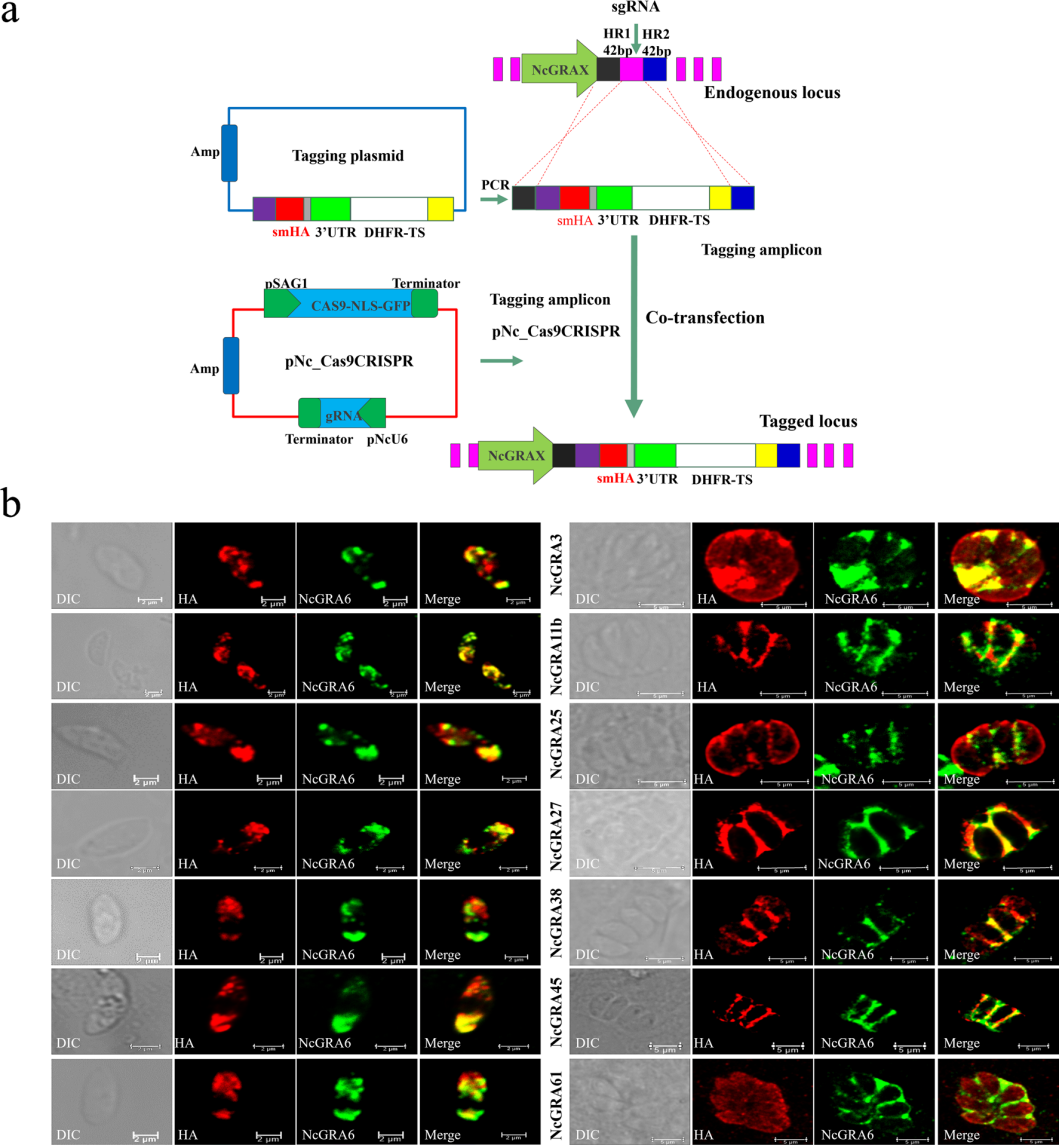
Identification of novel dense granule proteins by NcGRA17-BioID. **a** Schematic illustration of endogenous gene tagging at the C-terminus by CRISPR/Cas9-mediated site-specific insertion. Tagging plasmids were generated with smHA tag (red box) flanked by common ends (purple and yellow boxes) and including a common stop codon (gray box) followed by the HXGPRT 3′ untranslated region (UTR) (green box) and the selectable marker DHFR-TS. Amplification of this central region with primers that contained short homology regions HR1 (black box) and HR2 (blue box) together with the common flanks (purple and yellow boxes) generated products for gene-specific tagging. Co-transfection of these amplicons with a CRISPR/Cas9 plasmid bearing the gene-specific gRNA was used to add a smHA tag (red box) at the C-terminus of the endogenous locus. **b** IFA with rabbit anti-HA antibodies shows strong staining of the dense granules for each novel NcGRA that co-localizes with NcGRA6, demonstrating that these are novel dense granule proteins. Gene numbers and novel designations are as follows: NcLIV_227280, NcGRA3; NcLIV_012020, NcGRA11b; NcLIV_042680, NcGRA25; NcLIV_043760, NcGRA27; NcLIV_055980, NcGRA38; NcLIV_058760, NcGRA45; and NcLIV_057330, NcGRA61. (Intracellular, scale bar = 5 μm; extracellular, scale bar = 2 μm)

We selected a group of hypothetical proteins whose homologous protein in *Toxoplasma* had no signature cyclical expression pattern, and were constitutively expressed and highly expressed at the tachyzoite stage. To localize these candidates, we used CRISPR/Cas9 to add smHA epitope tags to the C termini (Fig. 2a; Additional file 2: Figure S2), as described previously [20]. The overexpression plasmid pDMG-HA was also used to add 3 × HA epitope tags to the C termini. Among our NcGRA17-BirA* hits, we epitope-tagged seven proteins that shared the same localization in extracellular parasites as NcGRA6: NCLIV_045870 (NcGRA3), NCLIV_012020 (NcGRA11b), NCLIV_042680 (NcGRA25), NCLIV_043760 (NcGRA27), NCLIV_055980 (NcGRA38), NCLIV_058760 (NcGRA45), and NCLIV_057330 (NcGRA61) (Fig. 2b). NcGRA3, NcGRA25, and NcGRA61 were located at the PVM, and the other proteins were located within the PV in intracellular parasites (Fig. 2b). Among the seven novel dense granule proteins, NcGRA3, NcGRA11b, NcGRA25, NcGRA38, and NcGRA45 have been previously described as orthologs in *Toxoplasma*.

### Identification and infectivity potential of NcGRA23

**Fig. 3:**
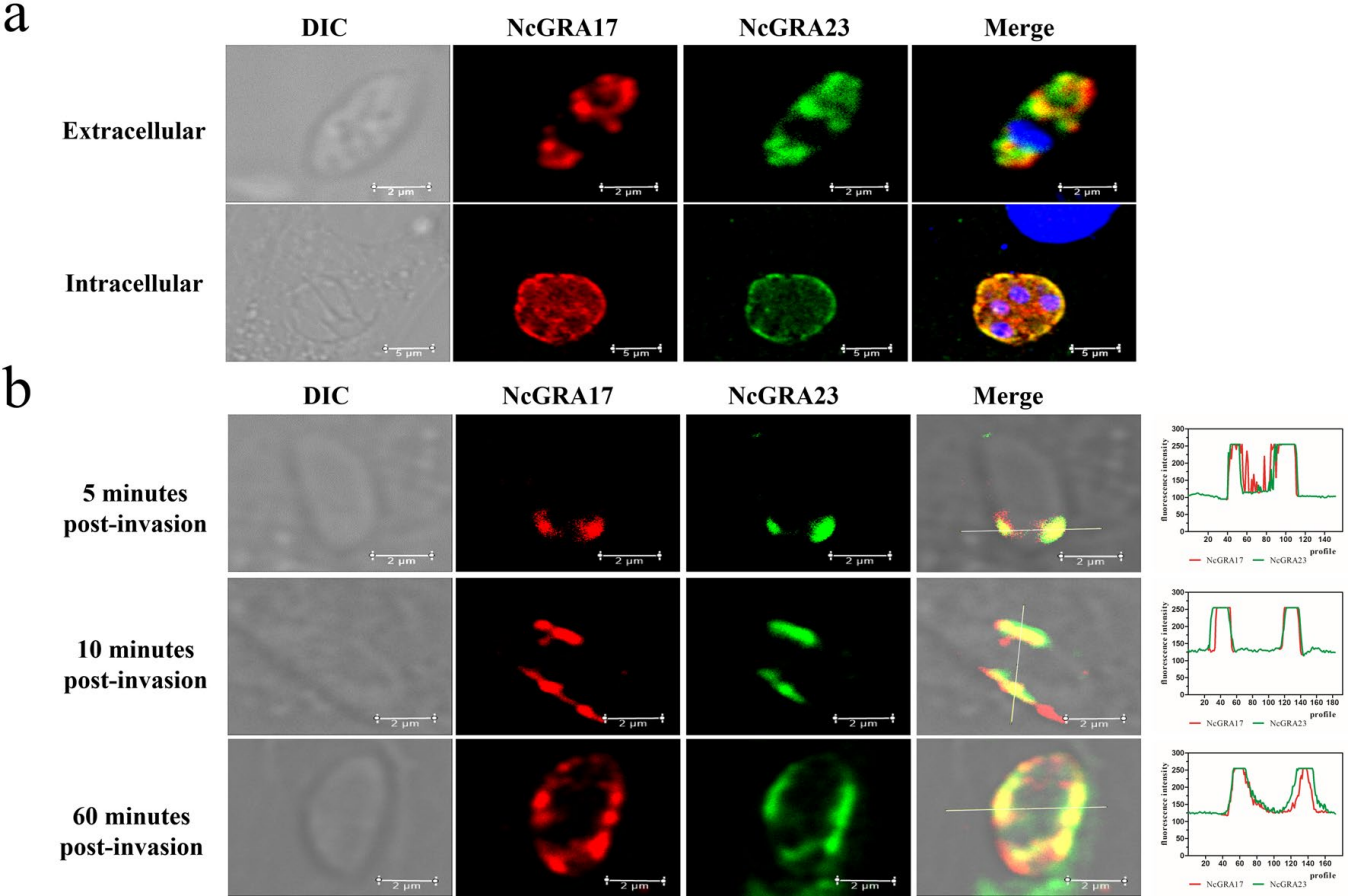
The localization of the NcGRA23. **a** IFA results showing that HA-tagged NcGRA17 co-localizes with NcGRA23 in dense granules and PVM (intracellular, scale bar = 5 μm; extracellular, scale bar = 2 μm). **b** Localization analysis of the distribution of NcGRA17-HA and NcGRA23 following secretion into the PV at 5 min, 10 min, and 1 h post-invasion (scale bar = 2 μm)

NcGRA23 was chosen for analysis because it was the closest in sequence similarity (47.8% amino acid sequence similarity) to NcGRA17 and scored highly in the mass spectrometry data set in our data (Table 1; Additional file 4: Table S2). To examine the subcellular localization, the full-length NcGRA23 protein fused with a histidine-tag was successfully expressed and used to generate anti-rNcGRA23 serum in mice. IFA analysis showed that the NcGRA23 protein of the parasites overlapped with NcGRA17 and the protein of intracellular tachyzoites was localized on the PVM (Fig. 3a). To determine the secretion characteristic of the protein, we also analyzed the secretion kinetics of the NcGRA23 at different time points (5 min to 1 h) after invasion. NcGRA23 was secreted into the PV at the apical end of the parasite following host cell invasion, consistent with the release of NcGRA17. At 10 min post-invasion, NcGRA23 was localized on both sides of the parasite, and then it was gradually dispersed throughout the vacuole by 60 min (Fig. 3b).

**Table 1:** Summary of known and novel NcGRAs found by NcGRA17-BioID

Gene ID (NcLIV no. [ToxoDB release 37])	Protein name	
006780	NcGRA23	
**057330**	**NcGRA61**	
045650	NcGRA2	
005560	NcGRA17	
**043760**	**NcGRA27**	
**045870**	**NcGRA3**	
**012020**	**NcGRA11b**	
**042680**	**NcGRA25**	
**055980**	**NcGRA38**	
**058760**	**NcGRA45**	

Novel NcGRAs are highlighted in boldface

**Fig. 4:**
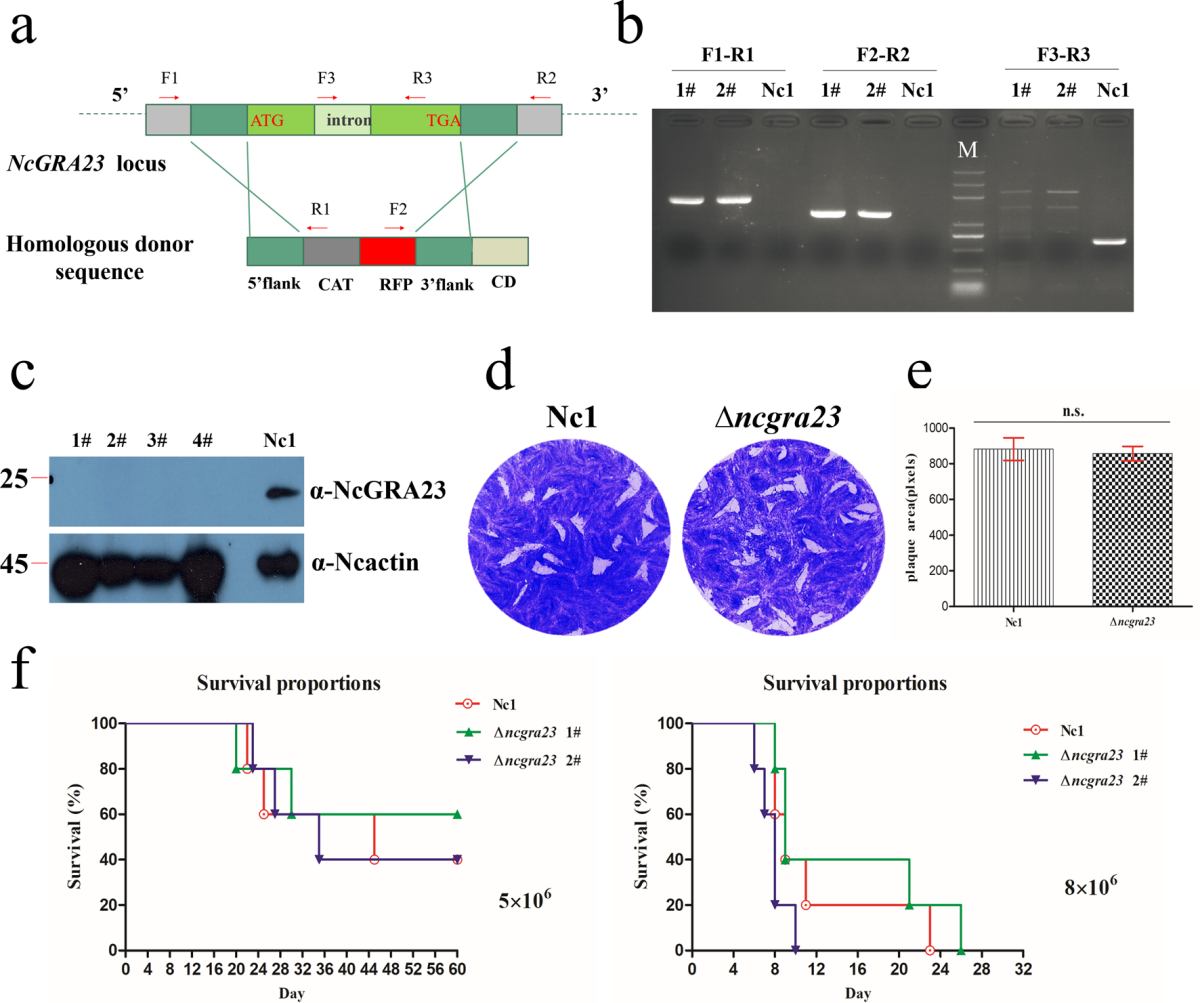
Infectivity potential of NcGRA23. **a** Schematic illustration of the replacement of the entire NcGRA23 coding region with the chloramphenicol resistance gene (*CmR*) and red fluorescence protein gene (*RFP*) for the generation of the *NcGRA23* knockout strain. **b** Diagnostic PCRs on two Δ*ncgra23* clones (1 and 2). (F1-R1) and (F2-R2) check the correct integration of the *NcGRA23* gene locus, whereas (F3-R3) examines the deletion of the *NcGRA23* gene. **c** Western blotting with NcGRA23 anti-mouse antibodies with NcGRA23 of 23 kDa in the parent Nc1 strain. No NcGRA23 protein was detected in the Δ*ncgra23* polyclonal strains (1, 2, 3, and 4). NcActin was used as a loading control. **d** Plaque assay comparing the growth of Nc1 and Δ*ncgra23*. Each well (HFF cell) was infected with 200 parasites, and plaques were stained 9 days later. **e** The plaque areas were counted by randomly selecting at least 20 plaques and using the pixel point in Photoshop C6Ssoftware (Adobe, USA). The data were compiled from two independent experiments. Analysis of the plaque area was performed using one-way ANOVA with Tukey’s post hoc comparison. n.s. indicates no difference (*p* > 0.05). **f** Mouse survival after infection with 5 × 10^6^ or 8 × 10^6^ doses of Δ*ncgra23* and Nc1. There were five female mice in each group. Statistical analysis was performed using the LIFETEST (life test data = surv) SAS software (SAS Institute Inc., USA). The data are representative of two experiments with similar outcomes

In *Toxoplasma*, the TgGRA17 and TgGRA23 proteins, which share amino acid sequence similarity with NcGRA17 and NcGRA23, function to facilitate the movement of small molecules or nutrients across the PVM [24]. NcGRA23 lacks a signal peptide, a transmembrane domain, and sequence motifs that suggest a functional role. To verify the biological role of *NcGRA23*, we successfully constructed the *NcGRA23* knockout strain (Fig. 4a). We isolated the *NcGRA23* knockout clones. Several strains with a complete knockout of the *NcGRA23* gene (∆*ncgra23*) were generated. PCR and western blotting confirmed the deletion of the *NcGRA23* gene (Fig. 4b, c). The growth of the *NcGRA23* gene knockout in the Nc1 strain was assayed by plaque formation on HFF monolayers as described above. The knockout mutant had no significant reductions in plaque formation (Fig. 4d, e). The knockout mutant was also examined for virulence in BALB/c mice. All of the mice inoculated with the wild-type or *NcGRA23* gene knockouts succumbed to infection with 8 × 10^6^ tachyzoites, with approximately equal time to death (Fig. 4f), and there was no significant difference in survival time between mice in the group infected with 5 × 10^6^ tachyzoites, indicating that this protein does not affect parasite virulence in mice.

### Identification and infectivity potential of NcGRA11

**Fig. 5:**
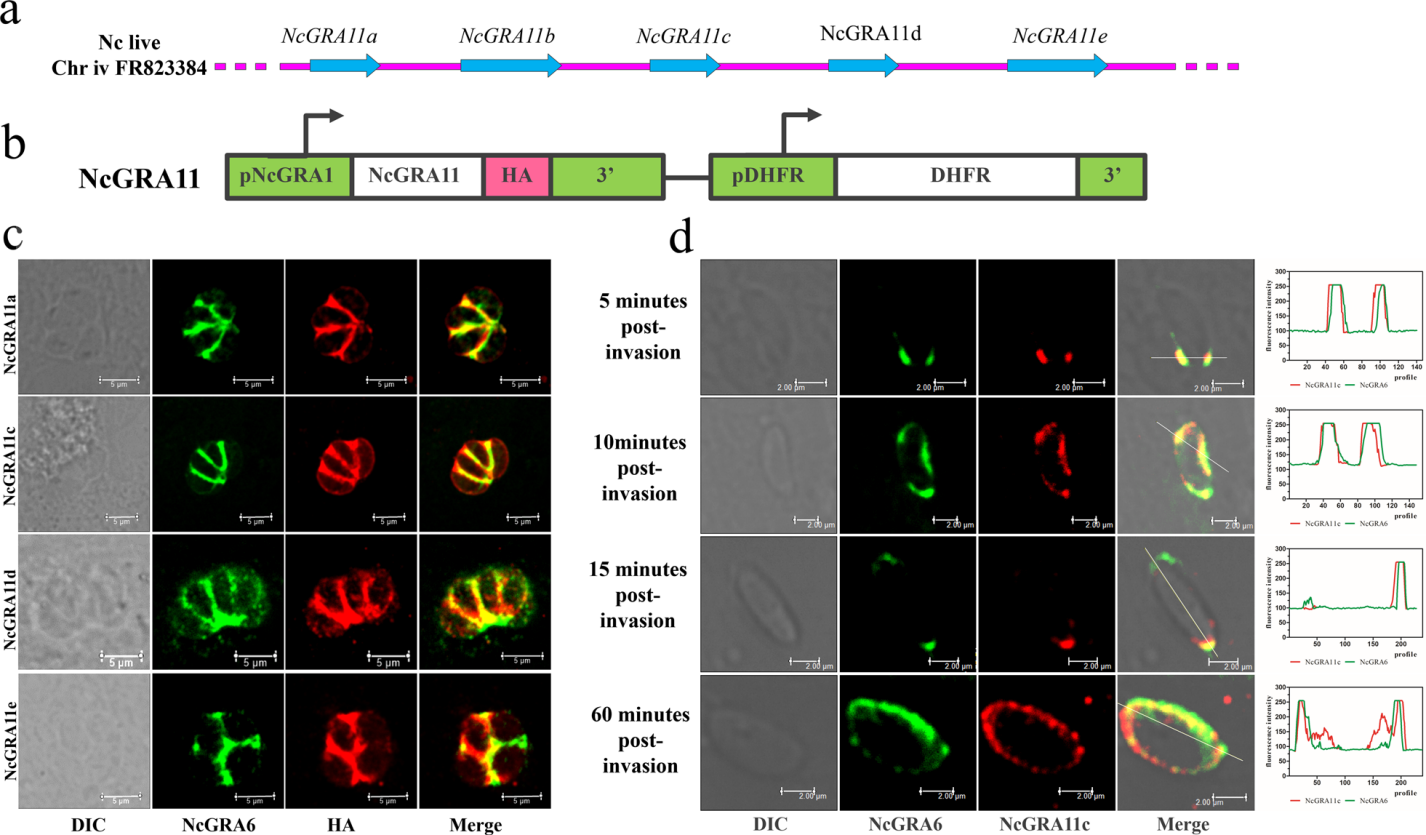
The localization of the NcGRA11 (a–e). **a** The localization of *NcGRA11 (a–e) *on the chromosome. **b** Schematic representation of the NcGRA1-NcGRA11-3 × HA-DHFR reporter constructs used for integration into the genome in Nc1 tachyzoites. **c** IFA results showing that HA-tagged NcGRA11 co-localizes with NcGRA6 in the PV (scale bar = 2 μm). **d** Localization analysis of the distribution of NcGRA11c-HA and NcGRA6 following secretion into the PV at 5 min, 10 min, 15 min, and 1 h post-invasion (scale bar = 2 μm)

Examination of novel identified dense granule proteins by BLAST analysis revealed four other proteins not found in the NcGRA17-BioID data set with similarity to NcGRA11b (NCLIV_012010, NCLIV_069640, NCLIV_012210, and NCLIV_012200). To verify the biological role of the four genes, we constructed the overexpression strains (Fig. 5b). The IFA results showed that proteins appeared to overlap with NcGRA6. Therefore, NCLIV_012010, NCLIV_069640, NCLIV_012210, and NCLIV_012200 were named NcGRA11a, NcGRA11c, NcGRA11d, and NcGRA11e, respectively (Fig. 5c). To determine the secretion characteristic of NcGRA11, we also analyzed the secretion kinetics of the NcGRA11c at different time points (5 min to 1 h) after invasion (Fig. 5d). At the earliest time when secretion was detected (5 min post-invasion), NcGRA11c-HA and NcGRA6 were secreted into the vacuole as concentrated foci on either side of the anterior part of the parasite. Following secretion into the vacuole, NcGRA11c and NcGRA6 were rapidly localized to the posterior end of the parasite cell (15 min post-invasion). By 60 min after invasion, NcGRA11c and NcGRA6 were found throughout the vacuole, forming an accumulation surrounding the parasite cell and with a distinct concentration at one pole. The patterns of secretion suggest that the exocytosed NcGRA11c and NcGRA6 may be rapidly translocated from the anterior to the posterior end to participate in network formation.

**Fig. 6:**
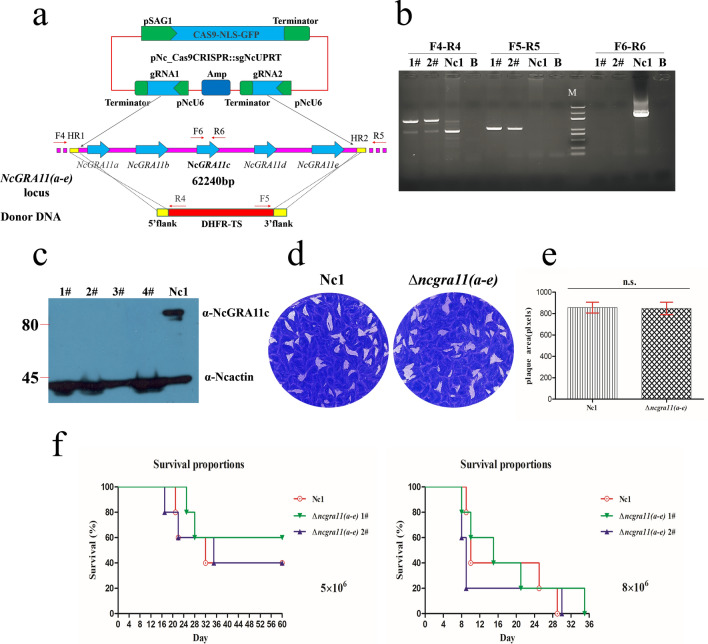
Infectivity potential of NcGRA11 (a–e). **a** Strategy for *NcGRA11 (a–e)* disruption by CRISPR/Cas9-mediated homologous gene replacement. **b** Diagnostic PCRs on two ∆*ncgra11 (a–e)* clones (1 and 2). (F4-R4) and (F5-R5) check the correct integration of the selection marker to the *NcGRA11 (a–e)* genes locus, whereas (F6-R6) examines the deletion of the *NcGRA11 (a–e)* genes. **c** Western blotting with NcGRA11c anti-mouse antibodies with NcGRA11c of 91 kDa in the parent Nc1 strain. No NcGRA11c protein was detected in the ∆*ncgra11 (a–e)* polyclonal strains (1, 2, 3, and 4). NcActin was used as a loading control. **d** Plaque assay comparing the growth of Nc1 and ∆*ncgra11 (a–e)*. Each well was infected with 200 parasites, and plaques were stained 9 days later. **e** The plaque areas were counted by randomly selecting at least 20 plaques and using the pixel point in Photoshop C6S software (Adobe, USA). The data were compiled from two independent experiments. Statistical analysis was performed as above. **f** Mouse survival after infection with 5 × 10^6^ or 8 × 10^6^ doses of ∆*ncgra11 (a–e)* and Nc1. There were five female mice in each group. Statistical analysis was performed as above

The five genes were then verified to be located together on chromosome 4 for 62,240 bp (Fig. 5a). The encoded proteins contain a predicted signal peptide and three transmembrane domains, and lack known functional domains. To generate clean knockouts of *NcGRA11 (a-e)* genes, a double-gRNA was used in targeting the 5′ and 3′ regions of the sequence to delete the entire sequence, followed by selection for insertion of the DHFR-TS selectable maker flanked by short, gene-specific homology regions (Fig. 6a), as described previously [17]. We were able to easily generate *NcGRA11 (a–e)* gene knockout strain (∆*ncgra11 (a-e)*), as shown using diagnostic PCR and western blotting (Fig. 6b, c). We investigated the replication and virulence of ∆*ncgra11 (a–e)*. The lack of NcGRA11 (a–e) did not affect parasite growth in vitro, as the plaque areas of ∆*ncgra11 (a–e)* were equivalent to Nc1 (Fig. 6d, e). We observed no significant difference in survival time between mice in the group infected with 5 × 10^6^ or 8 × 10^6^ tachyzoites of the different strains (Fig. 6f). The results above suggest that NcGRA11 (a–e) is not involved in parasite growth and replication.

## Discussion

*Neospora* dense granules are necessary storage secretory organelles. Today, the full proteome of dense granules remains unknown, and only a small number of NcGRA proteins (~ 9) have been identified. The analysis of protein content of dense granules is an important aspect of biological function study of *Neospora*. Biochemical fractionation approaches were used to isolate *Toxoplasma* dense granules, but the excessive parasite and/or host contamination limited its application [25]. Recently, the BioID technique has been used to screen proteins interacting with or in proximity to bait proteins, with high screening efficiency [20, 22, 26, 27]. Therefore, in this study, we adapted the BioID technique for *N. caninum* and demonstrated that BirA* fusions can biotinylate a robust number of proteins in the NcGRA.

We chose the dense granule protein NcGRA17 as bait for the initial BirA* fusion, because NcGRA17 is highly expressed and can be fused to an endogenous HA tag and traffic correctly to the dense granules [14]. Because NcGRA17 localized at the PV, we hypothesized that NcGRA17-BirA* should label proteins in the dense granules and PV. Indeed, our NcGRA17 BioID data set contains known and novel proteins from both sub-organelle compartments (see Table 1). The high success rate of our verifications by gene tagging supports specific labeling of NcGRAs secreted into the dense granules. We did identify eight NcGRA proteins within the PV and three NcGRA proteins associated with the PVM. The extent to which these are truly proteins that interact with NcGRA17 or merely proximal at the vacuolar remains to be determined.

Our study on the endogenous tagging and deletion of the NcGRAs coding sequences takes advantage of the improved efficiency of CRISPR/Cas9 for targeted gene editing in *Neospora* [14]. The target genes were rapidly tagged or deleted using a cassette approach where amplicons are generated from separate plasmids bearing distinct tags or resistance markers using a common set of primers containing short homology regions (42 or 60 bp) for gene targeting in the Nc1 background, as described in *Toxoplasma* previously [17]. The smHA tagging plasmids and resistance markers encoding DHFR plasmids described here are easily adapted to other genes of interest by designing new primer pairs and modifying the CRISPR/Cas9 plasmid or CRISPR/Cas9 double-gRNA plasmid **t**o contain different gRNAs, as described previously [28].

As a known *Neospora* dense granule protein, NcGRA23 was previously found to be localized at the PVM and shared sequence similarity with NcGRA17 [7, 14]. In agreement with this, NcGRA23 scored highly in the mass spectrometry data set (Table 1; Additional file 4: Table S2). In this study, NcGRA23 co-localized with NcGRA17 within the dense granules and at the PVM, and had same patterns of secretion with NcGRA17. To verify the biological role of NcGRA23, our attempts to disrupt the gene using CRISPR/Cas9 confirmed that NcGRA23 is nonessential for in vitro growth or in vivo virulence. Unfortunately, we were unable to generate a ∆*ncgra17*/∆*ncgra23* strain, which suggests that they may be essential for parasite growth. A tetracycline repressor-based system and plant-like auxin-induced degron (AID) system have been used in *Toxoplasma* essential gene study [20, 29]; these conditional gene knockout approaches may be used to explore the function of *Neospora* essential genes in the future.

While most of the dense granule proteins identified to date lack paralogs in *Neospora*, we identified a group of new NcGRA proteins (NcGRA11 a–e) that have shared sequence similarity. We then verified that the five genes were located together on chromosome 4 for 62,240 bp by amplifying and sequencing. It is noteworthy that proteins (NcGRA11 a–e) are significantly larger than most previously identified NcGRAs (~ 100 kDa versus the 30–50 kDa typical of most NcGRA proteins). It has been reported that TgGRA11B is a merozoite-specific protein that traffics into the PV and PVM [30]. We found that TgGRA11B and NcGRA11 (a–e) shared sequence similarity by sequence alignment. To assess the expression of NcGRA11 (a–e) in tachyzoites, we prepared a mouse anti-NcGRA11c polyclonal antibody and confirmed by western blotting that NcGRA11c is expressed in *Neospora* tachyzoites. However, the anti-rNcGRA11c serum failed to recognize the NcGRA11c protein in IFA, which meant the expression of the *NcGRA11c* gene might be low in the tachyzoite stage. The function of these new players was addressed by gene disruption, and this showed that these proteins are nonessential for parasite survival. NcGRA11 (a–e) may play roles during the coccidian or bradyzoite stages. However, this possibility needs to be examined in future studies.

## Conclusions

Our results highlight that biotinylation of proximal proteins using the BioID system is a powerful approach to rapidly identify novel NcGRA proteins. Using NcGRA17-BirA* fusion protein as bait, we have identified 11 novel GRA proteins. In addition, our findings indicate that NcGRA23 and NcGRA11 (a–e) are not involved in parasite propagation in vitro and virulence in vivo. The exact biological functions of novel identified GRA proteins need to be examined further in future studies.

## Supplementary Information

**Additional file 1: Figure S1.** Identification of the NcGRA17-BirA*-HA strains. a Schematic of the experimental design of the endogenous gene BirA*-HA tagging strain. CRISPR/Cas9-NcGRA17 was used to target the *NcGRA17* locus of Nc1 parasites. A donor vector (pLIC-BirA*-HA-DHFR-NcGRA17) was constructed. b Diagnostic PCR demonstrating homologous integration in parasites, compared with the parent line Nc1. F7-R7, F8-R8, and F9-R9 provide evidence of homologous integration based on products amplified between the donor sequence and regions in the *NcGRA17* locus that lie outside the targeting amplicon. c Western blotting showing NcGRA17 with the predicted size of 66.3 kDa.

**Additional file 2: Figure S2.** Verification of endogenous gene tagged with smHA. PCR products of NcGRA3-HA (a), NcGRA25-HA (b), NcGRA27-HA (c), NcGRA38-HA (d), NcGRA45-HA (e) and NcGRA61-HA (f) shown confirming the endogenous gene smHA tagging (primer numbers refer to the primers found on Additional file 3: Table S1). *Nc5* gene served as a *N. caninum* specific gene.

**Additional file 3: Table S1.** Primers used in this study.

**Additional file 4: Table S2.** Mass spectrometry results for the identification of proteins from NcGRA17-BirA* based biotinylation.

**Additional file 5: Table S3.** Human protein hits from NcGRA17-BioID mass spectrometry.

## Data Availability

All data analyzed or generated during this study are included in this published article.

## Abbreviations

*N. caninum*: *Neospora caninum*
GRA: Dense granule
GRAs: Dense granule proteins
NcGRAs: *Neospora* dense granule proteins
TgGRAs: *Toxoplasma* dense granule proteins
PV: Parasitophorous vacuole
BioID: Biotin identification
PVM: Parasitophorous vacuole membrane
MNN: Membranous nanotubular network
DMEM: Dulbecco’s modified Eagle’s medium
HFF: Human foreskin fibroblast
UHPLC: Ultrahigh-pressure liquid chromatography
MS: Mass spectrometry
NSAF: Normalized spectral abundance factor
AID: Auxin-induced degron
