# Supplementary figures and images for: Biotinylation of the Neospora caninum parasitophorous vacuole reveals novel dense granule proteins
Source: Parasit Vectors. 2021 Oct 9;14:521. doi: 10.1186/s13071-021-05023-7 (PMC8501707; doi:10.1186/s13071-021-05023-7)

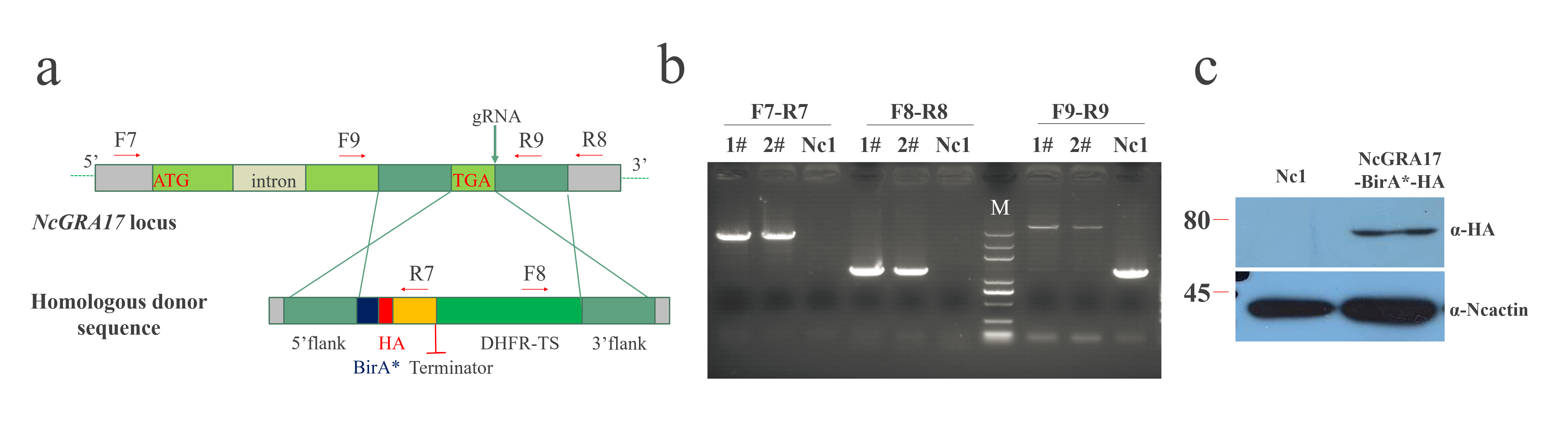

Supplement: Supplementary file 1 — Additional file 1: Figure S1. Identification of the NcGRA17-BirA*-HA strains. a Schematic of the experimental design of the endogenous gene BirA*-HA tagging strain. CRISPR/Cas9-NcGRA17 was used to target the NcGRA17 locus of Nc1 parasites. A donor vector (pLIC-BirA*-HA-DHFR-NcGRA17) was constructed. b Diagnostic PCR demonstrating homologous integration in parasites, compared with the parent line Nc1. F7-R7, F8-R8, and F9-R9 provide evidence of homologous integration based on products amplified between the donor sequence and regions in the NcGRA17 locus that lie outside the targeting amplicon. c Western blotting showing NcGRA17 with the predicted size of 66.3 kDa. [file 13071_2021_5023_MOESM1_ESM.tif]

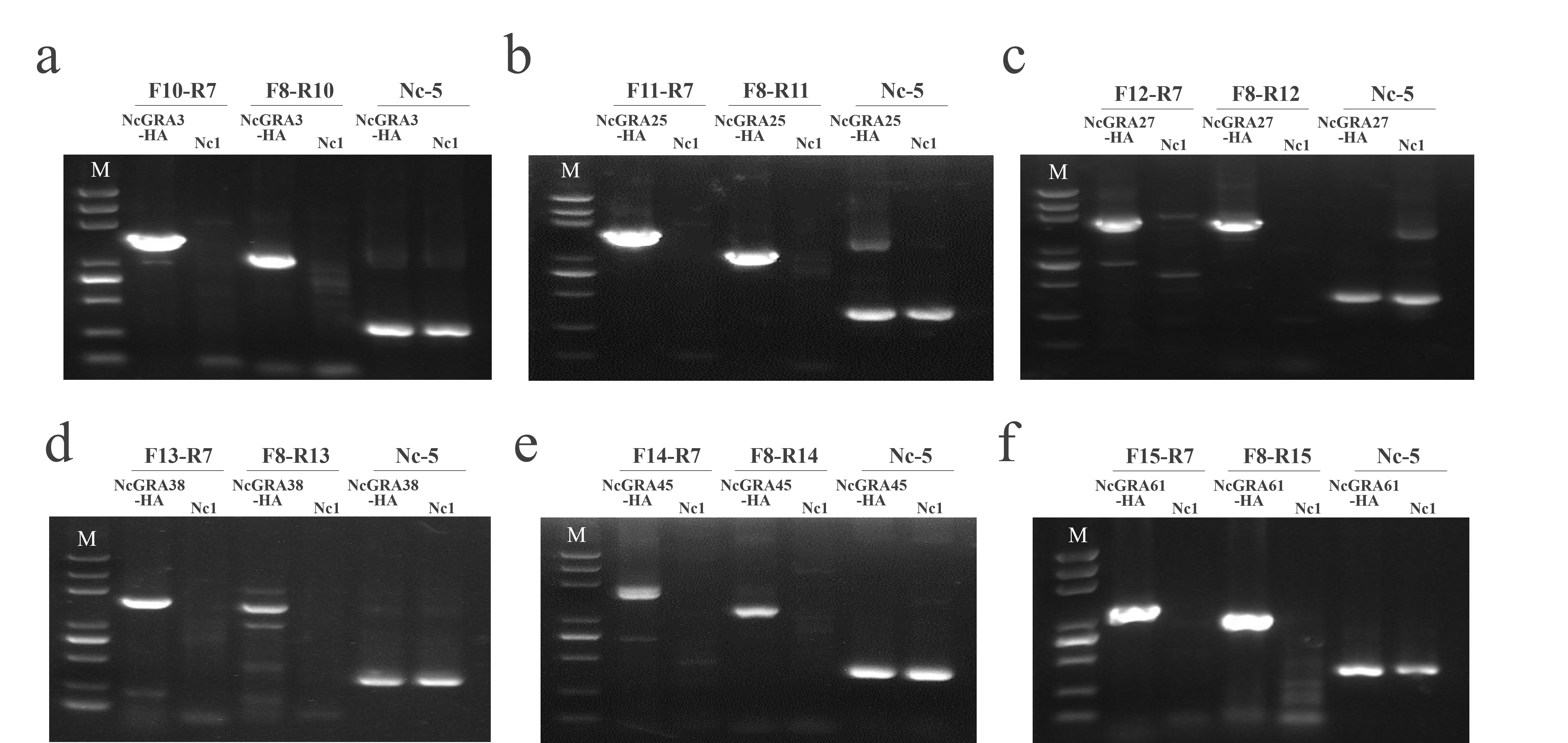

Supplement: Supplementary file 2 — Additional file 2: Figure S2. Verification of endogenous gene tagged with smHA. PCR products of NcGRA3-HA (a), NcGRA25-HA (b), NcGRA27-HA (c), NcGRA38-HA (d), NcGRA45-HA (e) and NcGRA61-HA (f) shown confirming the endogenous gene smHA tagging (primer numbers refer to the primers found on Additional file 3: Table S1). Nc5 gene served as a N. caninum specific gene. [file 13071_2021_5023_MOESM2_ESM.tif]
