# Supplementary material for: Biotinylation of the Neospora caninum parasitophorous vacuole reveals novel dense granule proteins
Source: Parasit Vectors. 2021 Oct 9;14:521. doi: 10.1186/s13071-021-05023-7 (PMC8501707; doi:10.1186/s13071-021-05023-7)
Supplement: Supplementary file 3 — Additional file 3: Table S1. Primers used in this study. [file 13071_2021_5023_MOESM3_ESM.doc]

Biotinylation of the *Neospora caninum* Parasitophorous Vacuole Reveals Novel Dense Granule Proteins

Congshan Yang^1, 2^, Chenrong Wang^1^, Jing Liu^1^, Qun Liu^1^**^*^**

Table S1. Primers used in this study.

| Primer name | Sequences | enzyme  sites |
| --- | --- | --- |
| F1 | 5′- TGTGGTTTTCCCAAATAGT -3′ |  |
| R1 | 5′- CAACTGACTGAAATGCCTC -3′ |  |
| F2 | 5′- CAACCACAACGAGGACTACAC -3′ |  |
| R2 | 5′- TAGACCCGCAAAGATACTGAT -3′ |  |
| F3 | 5′- ACGGGTACGAGCAAACAAAT -3′ |  |
| R3 | 5′- GAAGAGCAGATCAGCCATCA -3′ |  |
| F4 | 5′- GCGGCGCCCTCTAAGTGTCGCAC -3′ |  |
| R4 | 5′- TTGCGGTGTCGTGGATTTAC -3′ |  |
| F5 | 5′- ACAGTCTCACCTCGCCTTGT -3′ |  |
| R5 | 5′- GAATTCTCATCCTCCCTATTTC -3′ |  |
| F6 | 5′- ATGTCCCGTCGTTTGGCATC -3′ |  |
| R6 | 5′- CTCTCCCTTGATATTCTTTT -3′ |  |
| F7 | 5′- ATGCGAGTGTGCGGTTCCTT -3′ |  |
| R7 | 5′- GGACAGAACAATACCGGCACCA -3′ |  |
| F8 | 5′- ATCCACTCGTGAATGCGTTAT -3′ |  |
| R8 | 5′- AAAGGGCCTCAAGGCACTCTA -3′ |  |
| F9 | 5′- CCTGGGCTTACGGGGCTTTA -3′ |  |
| R9 | 5′- GTTGGTTGGCACGCGCACAT -3′ |  |
| F10 | 5′- TTAGCGGCTATGGGCAAGAG -3′ |  |
| R10 | 5′- ACAATGGCACGGAACAAACG -3′ |  |
| F11 | 5′- AGAGAGCGCAGCAGTTTCTT -3′ |  |
| R11 | 5′- TCAGATACAGTGCATCCCGC -3′ |  |
| F12 | 5′- TGTCGGCTGAACTAATCTG-3′ |  |
| R12 | 5′- TGAAGTCGAGGAACGGAATT -3′ |  |
| F13 | 5′- CGCGTGTCTGTCACTTTTGG -3′ |  |
| R13 | 5′- CGAGCCTTAACCGCACAAAG -3′ |  |
| F14 | 5′- AGGAAGATGTCCGCAATGGG-3′ |  |
| R14 | 5′- ACAACGAATGACGCTGACCT -3′ |  |
| F15 | 5′- CGATTAGCAGTGGTGGCGAT-3′ |  |
| R15 | 5′- TCTGTTCCTGGGCCGATCTA -3′ |  |
| Cas9 F | 5′- ATACGACTCACTATAGGGCG -3′ |  |
| Cas9 R | 5′- AGCTCCACCGCGGTGGCGGC -3′ |  |
| NcU6-gRNA F | 5′- GCCGCCACCGCGGTGGAGCTGAGGGCAAAACTCCACAAA -3′ |  |
| gRNA-AMP R | 5′- CGCCCTATAGTGAGTCG -3′ |  |
| NcGRA17-gRNA-AMP F | 5′- CCAGTGAGCGGCCCGACAGTGTTTTAGAGCTAGAAATAG -3′ |  |
| NcU6- NcGRA17-gRNA R | 5′- ACTGTCGGGCCGCTCACTGGAAACAACAATGTCCCTTTG -3′ |  |
| NcGRA3-gRNA-AMP-F | 5′- GCATTTTGTTTTCGACCTCGGTTTTAGAGCTAGAAATAG-3′ |  |
| NcU6-NcGRA3-gRNA-R | 5′- CGAGGTCGAAAACAAAATGCAAACAACAATGTCCCTTTG-3′ |  |
| NcGRA25-gRNA-AMP-F | 5′- GCATCCACAAACTCTGAGTAGTTTTAGAGCTAGAAATAG-3′ |  |
| NcU6-NcGRA25-gRNA-R | 5′- TACTCAGAGTTTGTGGATGCAAACAACAATGTCCCTTTG-3′ |  |
| NcGRA38-gRNA-AMP-F | 5′- GGTCCGGGAGAGTCCCTATGGTTTTAGAGCTAGAAATAG-3′ |  |
| NcU6-NcGRA38-gRNA-R | 5′- CATAGGGACTCTCCCGGACCAAACAACAATGTCCCTTTG-3′ |  |
| NcGRA47-gRNA-AMP-F | 5′- GATGCCGATTTAATTTTCTTGTTTTAGAGCTAGAAATAG-3′ |  |
| NcU6-NcGRA47-gRNA-R | 5′- AAGAAAATTAAATCGGCATCAAACAACAATGTCCCTTTG-3′ |  |
| NcGRA61-gRNA1-AMP-F | 5′- GCAATCGTTGCACGTCTTGCGTTTTAGAGCTAGAAATAG-3′ |  |
| NcU6-NcGRA61-gRNA-R | 5′- GCAAGACGTGCAACGATTGCAAACAACAATGTCCCTTTG-3′ |  |
| NcGRA26a-gRNA-AMP-F | 5′- GTGCGCCCGAATGCTCTGTGGTTTTAGAGCTAGAAATAG-3′ |  |
| NcU6-NcGRA26a-gRNA-R | 5′- CACAGAGCATTCGGGCGCACAAACAACAATGTCCCTTTG-3′ |  |
| NcGRA26b-gRNA-AMP-F | 5′- GCGTCCCTGTACGCCCGTATGTTTTAGAGCTAGAAATAG-3′ |  |
| NcU6-NcGRA26b-gRNA-R | 5′- ATACGGGCGTACAGGGACGCAAACAACAATGTCCCTTTG-3′ |  |
| NcGRA27-gRNA-AMP-F | 5′- GTAACGTATAATCCTCGCCGGTTTTAGAGCTAGAAATAG-3′ |  |
| NcU6-NcGRA27-gRNA-R | 5′- CGGCGAGGATTATACGTTACAAACAACAATGTCCCTTTG-3′ |  |
| NcGRA45-gRNA-AMP-F | 5′- GCCGTTTTGCAGTGACCAGAGTTTTAGAGCTAGAAATAG-3′ |  |
| NcU6-NcGRA45-gRNA-R | 5′- TCTGGTCACTGCAAAACGGCAAACAACAATGTCCCTTTG-3′ |  |
| NcGRA11(a-e)-gRNA1-AMP-F | 5′- GGCGTCCCACGGCGCAGCTTGTTTTAGAGCTAGAAATAG-3′ |  |
| NcU6-NcGRA11(a-e)-gRNA1-R | 5′- AAGCTGCGCCGTGGGACGCCAAACAACAATGTCCCTTTG-3′ |  |
| NcGRA11(a-e)-gRNA2-AMP-F | 5′- TAGTAGGCCGTCAGCGGACTGTTTTAGAGCTAGAAATAG-3′ |  |
| NcU6-NcGRA11(a-e)-gRNA2-R | 5′- AGTCCGCTGACGGCCTACTAAAACAACAATGTCCCTTTG-3′ |  |
| 2×gRNA backbone F1 | 5′- CGAGGTCGACGGTATCGATA -3′ |  |
| 2×gRNA backbone R1 | 5′- AGCTCCACCGCGGTGGCGGC -3′ |  |
| 2×gRNA backbone F2 | 5′- GCCGCCACCGCGGTGGAGCT -3′ |  |
| 2×gRNA backbone R2 | 5′- CGCCCTATAGTGAGTCG -3′ |  |
| 2×gRNA NcU6 F | 5′- TACGACTCACTATAGGGCGGAGGGCAAAACTCCACAAAA -3′ |  |
| 2×gRNA NcU6 R | 5′- TATCGATACCGTCGACCTCGCACAGGAAACAGCTATGAC -3′ |  |
| NcGRA3-HR1-F | 5′- AAGAAAAAGGGGAAAAAAGCCTCTCGCAAGCACGAAAGAAATGCTAGCAAGGGCTCGG-3′ |  |
| NcGRA3-HR2-R | 5′- GTGCGACGCCGGTAAATACCCGCGTAAACAATGACACCTCGAATACGACTCACTATAGG-3′ |  |
| NcGRA25-HR1-F | 5′- GAACCTCGACGAGAAAAGAAGAATCTTCAACAAACTCGTCGTGCTAGCAAGGGCTCGG-3′ |  |
| NcGRA25-HR2-R | 5′- TGTGCGGAGCTCCGTCTGGATCCCCGGTCCTGCTCTCCGTACATACGACTCACTATAGG-3′ |  |
| NcGRA38-HR1-F | 5′- GACTTTGCATTCACGCTCCCAGAGTGCAATCCTTTCGATGCCGCTAGCAAGGGCTCGG-3′ |  |
| NcGRA38-HR2-R | 5′- TTCACGTATTCGAACACGTATGATACAGCTAAGCGTCCACATATACGACTCACTATAGG-3′ |  |
| NcGRA47-HR1-F | 5′- TTTTTGTTCTACAAGGCAAAAAACTGGAAAAAGTCAAGAAATGCTAGCAAGGGCTCGG-3′ |  |
| NcGRA47-HR2-R | 5′- CCGATTTGACACACGCGCGACAAACAAGCCTTAAATCCGAAGATACGACTCACTATAGG-3′ |  |
| NcGRA61-HR1-F | 5′- GACGGATCGTCAGACGACGATCGCGACGTCCGAAAAAAATCAGCTAGCAAGGGCTCGG-3′ |  |
| NcGAR61-HR2-R | 5′- ATGTTGCCTTCTGTCAAATGAATTCCGGCCTCCATTCCTGCAATACGACTCACTATAGG-3′ |  |
| NcGRA26a-HR1-F | 5′- AAAGCTCAGCGGCGTGCGGTTGTGAGAGATACAGACGGTTCTGCTAGCAAGGGCTCGG-3′ |  |
| NcGRA26a-HR2-R | 5′- CTATCAAGGTGGAAGGTAGCAAGCGACGGTTTGCTCCCACACATACGACTCACTATAGG-3′ |  |
| NcGRA26b-HR1-F | 5′- AAAGCTCAGCGACGTGCGGTGGTGAGGGGCACATACGTTCCTGCTAGCAAGGGCTCGG-3′ |  |
| NcGRA26b-HR2-R | 5′- TCCCCCAGGCTTCTGTCAGAACGTCACATCGAATACCCGATAATACGACTCACTATAGG-3′ |  |
| NcGRA27-HR1-F | 5′-GCAGTTGGTGTCATTACATTTATTGGTTTTTTGTCTTCCGTTTTTGCGAGCATGCACTCTGCTAGCAAGGGCTCGG-3′ |  |
| NcGRA27-HR2-R | 5′-TCATGAACTTCCCCGCCCCGCTAAGCGGAATCAAACGTGTCACCTCACTAATCACCTCGGATACGACTCACTATAGG-3′ |  |
| NcGRA45-HR1-F | 5′- GATTCCGGCGCAGCTGCTTTCGACATGTTAGTTAAGGAGCATGCTAGCAAGGGCTCGG-3′ |  |
| NcGRA45-HR2-R | 5′- TAAAAATGCGAGAGAATGCGATCGCCCCGCTCCACACCGTCTATACGACTCACTATAGG-3′ |  |
| NcGRA11(a-e)-DHFR HR1-F | 5′-TGGCAGAAAATCCCGCTGAAGAGTTATCTCGCACCGGTCGTCTGGCGTCCCACGGCGCAGCTAGCATGTCATTCGATTTT-3′ |  |
| NcGRA11(a-e)-DHFR HR2-R | 5′-AGTGTGGAAAGCGTTTGTCGACTCAGCGGACAGGAAACACAGTTGACGCTCATGCCGAGTACTAGTGGATCGATCCCCCG-3′ |  |
| NcGRA11a-F | 5′- ATGTCCCGTCGTTTGGCATCCA-3′ |  |
| NcGRA11a-R | 5′- CTCTCCCTTGATATTCTTTTCC-3′ |  |
| NcGRA11b-F | 5′- ATGTGTCGAGGAGGCAAAAATG-3′ |  |
| NcGRA11b-R | 5′- CTCTCCCTTGATATTCTTTTCC-3′ |  |
| NcGRA11c-F | 5′- ATGCGTCGAGGAAGCGAAAACT-3′ |  |
| NcGRA11c-R | 5′- GTCACTAGTGGCGAATATCTGC-3′ |  |
| NcGRA11d-F | 5′- ATGCGTCGAGGAAGCGAAAACT-3′ |  |
| NcGRA11d-R | 5′- ATCATTTTTTGGTGCCTGCAGC-3′ |  |
| NcGRA11e-F | 5′- ATGCATCGAGGAGGCAAAAATG-3′ |  |
| NcGRA11e-R | 5′- GCTGACGTGATGCTGAGTCTGC-3′ |  |
| NcGRA17 (BirA*-HA)-5’flank F | 5′- gtccGATATCCCTGGGCTTACGGGGCTTTA -3′ | **EcoRV** |
| NcGRA17 (BirA*-HA)-5’flank R | 5′- gacgCCTAGGCTGGTTGCCACTGCCGGATC -3′ | **AvrⅡ** |
| NcGRA17 (BirA*-HA)-3’flank F | 5′- GCAGCCAGTGCGAAGAAGGG -3′ |  |
| NcGRA17 (BirA*-HA)-3’flank R | 5′- CGAATACACTAGTACATGGA -3′ |  |
| NcGRA23-5’flank F | 5′- GCAGACTCTGTCACGGTACT -3′ |  |
| NcGRA23-5’flank R | 5′- TGTCCCGGCGATGGCAGCAA -3′ |  |
| NcGRA23-3’flank F | 5′- gtccGATATCTGCTTGCGTCCAGTGTTCAT -3′ | **EcoRV** |
| NcGRA23-3’flank R | 5′- gacgCCTAGGAACCCTTTCGGCAATCATAT -3′ | **AvrⅡ** |
| pTCR-X-CD Linearize F | 5′- CAAGGCGATTAAGTTGGGTA -3′ |  |
| pTCR-X-CD Linearize R | 5′- TGTGGAATTGTGAGCGGATA -3′ |  |
| pLIC-BirA*-HA-X Linearize F | 5′- CAGGAAACAGCTATGACC -3′ |  |
| pLIC-BirA*-HA-X Linearize R | 5′- TGTAAAACGACGGCCAGT -3′ |  |
| rNcGRA23-F | 5′-ccgGAATTCATGCTCGCGTCCGCCGACGAAGCTT -3′ | **EcoR I** |
| rNcGRA23-R | 5′-ccgTCGAGTTAGTTCTTTCGCGCGAGCACTGACT -3′ | **Xhol I** |
| rNcGRA11c-F | cgGAATTCATGAGACAACAGCAAGGGGAA | **EcoRI** |
| rNcGRA11c-R | ataagaatGCGGCCGCGGCTTGCGCCTCGGACGCCTTG | **NotI** |

|  |
| --- |

|  |  |
| --- | --- |
